# Scooter-Driving Humanoid Robot: Sim-to-Real Transfer Through Deep Reinforcement Learning

**DOI:** 10.3390/s26196324

**Published:** 2026-10-07

**Authors:** Ugo Richard Roux, Saeed Saeedvand, Jacky Baltes

**Affiliations:** Educational Robotics Center, Department of Electrical Engineering, National Taiwan Normal University, Taipei 106, Taiwansaeedvand@ntnu.edu.tw (S.S.)

**Keywords:** humanoid robots, reinforcement learning, sim-to-real transfer, domain randomization, vehicle control, proximal policy optimization, inverse kinematics

## Abstract

This paper presents a simulation-to-reality (sim-to-real) transfer process, allowing a full-sized humanoid robot to autonomously balance a two-wheeled scooter and track operator-supplied heading commands through a deep reinforcement learning (DRL) policy, including while carrying a human passenger for the first time. The learned policy governs the coupled balance and steering dynamics only: the heading reference and the target speed are supplied by a human operator, the throttle is set externally, and the initial launch over the first 2 to 3 m and the final stop are performed manually for safety. The system therefore performs autonomous balance and steering-command tracking during motion, rather than autonomous scooter driving. Unlike four-wheeled vehicles where the inherent stability simplifies control, scooter operation demands continuous and precise dynamic balancing coupled with real-time steering control, creating a challenging full-body control task. In this study, we demonstrate a successful integration of DRL techniques to bridge the sim-to-real gap, achieving stable closed-loop control of a humanoid robot balancing and steering a scooter under significant model uncertainty. A multi-scenario real-world evaluation shows the system maintaining control in the demanding low-speed regime of 0.8 to 3.0 ${m\;s}^{-1}$, where balance control is most challenging and where speed varied across the range during trials rather than being held at a set point. Success rates are 0.96 on straight-line driving, 0.92 and 0.72 on light and sharp turns, 0.88 on the traversal of a 60 mm speed bump, and 0.73 while carrying a 70 kg passenger. They fall where sustained precise tracking is required, to 0.28 on a 4.5 m roundabout and 0.33 on part of the standardized Taiwanese scooter license test.

## 1. Introduction

As humanoid robotics technology matures and starts to reach the general consumer, the focus is shifting from specialized industrial automation to the consumer market, where service humanoid robots are expected to integrate seamlessly into daily human life. A primary barrier to widespread consumer adoption is the incapacity of current robots to utilize existing human-centric infrastructure and unmodified consumer tools. Rather than redesigning every household object or vehicle into a specialized device, next-generation consumer robots must possess the versatility to operate standard equipment used by humans themselves. Recent advances in robot control show success in multiple areas, such as humanoid robot walk control [1], locomotion tasks [2], household tasks [3], and factory manipulation applications [4]. However, extending these capabilities to dynamic interaction with complex mobility tools remains an open challenge. Controlling two-wheeled vehicles presents unique challenges due to inherent system instability and coupling between balance and steering, requiring real-time control closer to what is currently seen in locomotion tasks. This difficulty is amplified in our setup: the robot has no roll degree of freedom to control its upper body, and therefore cannot lean into turns. Unlike a human rider, who leans their body into turns to shift the center of mass and assist balance, the robot must stabilize and steer the scooter through steering control alone, which substantially narrows the available control authority. In this study, we introduce a multi-purpose humanoid robot that, in addition to conventional robotics challenges [5,6,7,8], can ride a common scooter, rather than modifying it into an autonomous controlled device, or applying traditional control-based approaches.

It is worth stating why the objective is a humanoid riding an unmodified scooter rather than a scooter automated directly. Automating the scooter is a vehicle-specific problem: the resulting controller transfers to no other machine. Posing the task as a humanoid operating the standard handlebar-and-seat interface instead keeps the control problem defined in terms of the human form, which is the interface that existing vehicles and tools are already built around. This is the motivation for the task rather than a result of it: the present work demonstrates sim-to-real transfer for a single humanoid–scooter pair, and does not test whether the learned skill transfers across other tools, vehicles, or robot morphologies.

Riding a scooter poses significant simulation-to-reality (sim-to-real) challenges due to multiple ill-defined subsystems that are difficult to model accurately. These include wheel shapes, system inertia and mass distribution, seat cushion flexibility, oscillations from actuator movement, scooter frame characteristics and flexibility, and robot–scooter contact points. These uncertainties complicate accurate simulation of real-world conditions and motivate the choice of reinforcement learning (RL) over classical model-based approaches. This learning-based approach allows a better adaptation to real-world uncertainties without requiring precise system identification, a critical advantage when deploying general-purpose robots to operate diverse, unmodeled human tools. Preliminary attempts at classical PID and model-predictive control did not achieve stable control under these unmodeled dynamics; we report them here only as motivation rather than as formally tuned baselines. As the first system to demonstrate this task, this work is intended to establish a baseline for our own future work rather than to compete against an existing solution, and we accordingly evaluate the system through component ablations.

The novelty of this work lies at the system and sim-to-real level rather than in the underlying learning algorithm. We deliberately build on established, well-understood components (PPO and domain randomization) so that the contribution can be attributed to the problem formulation and transfer methodology rather than to algorithmic tuning; introducing a new optimizer would confound, rather than clarify, what makes this previously undemonstrated task feasible. The contribution is therefore the first physical realization of a full-sized humanoid operating an unmodified commercial scooter, together with the system design that makes it possible: an inverse-kinematics formulation that decouples steering from throttle on a closed-loop hand–handlebar coupling that the simulator cannot represent, and a domain-randomization and symmetry-augmentation strategy tailored to the humanoid–scooter system. In continuation of our previous simulation-based study [9], we address the unique and challenging dynamic control problem of enabling a full-sized humanoid robot to autonomously balance a scooter and track heading commands in the real world (a video demonstration of the experimental results is available at https://youtu.be/39-NBm2o5u4 (accessed on 29 September 2026)). Our main contributions are as follows:

- Achieving stable steering control in the challenging low-speed regime of 0.8 to 3.0 ${m\;s}^{-1}$, where two-wheeled balance is hardest, while navigating over uneven surfaces.
- A multi-scenario real-world evaluation on the physical robot, including standardized Taiwanese scooter license test maneuvers and challenging scenarios (e.g., speed bumps, carrying passengers).
- A sim-to-real strategy specifically developed for our humanoid–scooter system, with diverse domain randomization including seating position variation, control delay parameters, and robot–scooter coupling uncertainties.
- Implementation of a sim-to-real transfer solution, enabling a full-sized humanoid robot to autonomously control the balance and steering of a two-wheeled scooter under operator-supplied heading and speed commands, with the ability to carry a human passenger in the presence of significant system uncertainty for the first time.
- A complete system architecture that integrates RL-based steering control and a specific reward function with total body control for humanoid vehicle operation.

**Figure 1:**
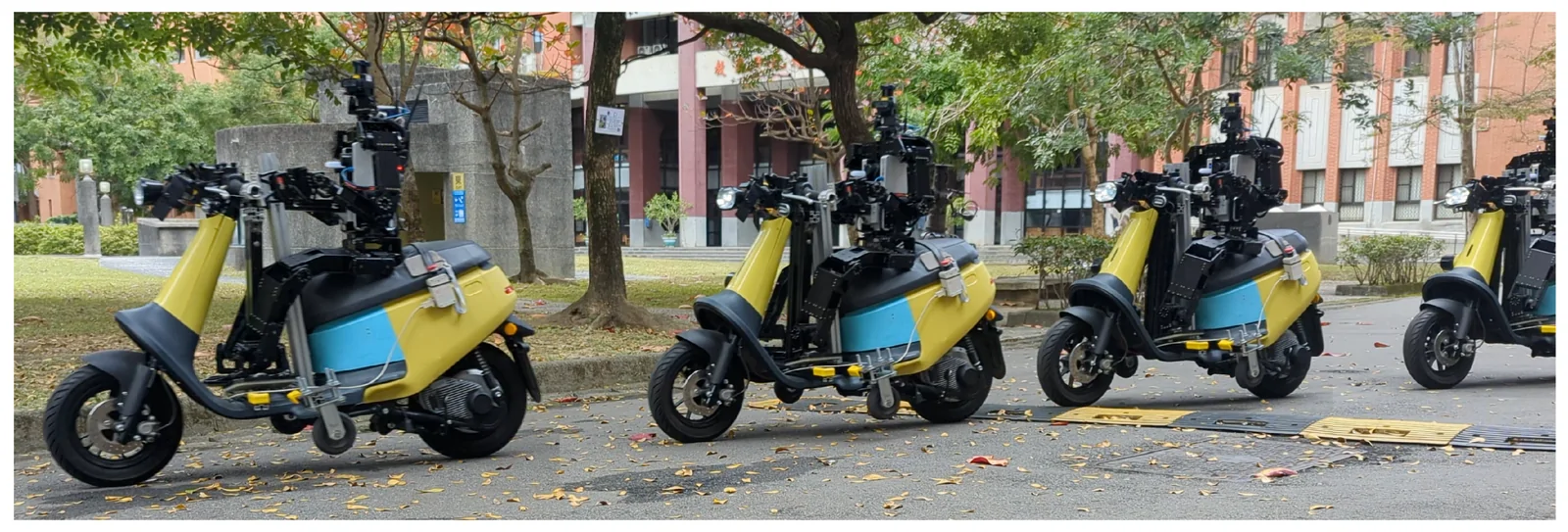
Robot–Scooter system passing a bumper at 1.6 ${m\;s}^{-1}$.

We demonstrate that proximal policy optimization (PPO) [10] and domain randomization [11] are able to learn a controller that can successfully bridge the sim-to-real gap for this task when appropriately integrated into a complete system architecture despite serious modeling limitations. Figure 1 shows the robot–scooter system crossing a speed bump, one of the balance control problems addressed in this study.

The rest of the paper is organized as follows. In Section 2, we give a brief literature review of balance control problems and highlight the current gap in the literature. Section 3 explains the different methods used to close the sim-to-real gap. The experimental setup is described in Section 4 and the results of the experiments are discussed in Section 5. Section 6 sets out the limitations of the study and Section 7 concludes.

## 2. Related Work

### 2.1. General Humanoid Robots

The recent push toward realistic human environment-capable humanoid robots reflects a trend for platforms not only able to perform stable bipedal locomotion on different surfaces [1,12] but also capable of everyday household tasks [3,5] and factory manipulation tasks [2,4]. These involve interaction with human-centric machinery and infrastructure to which the robot must adapt. Humanoid robots have also been shown to operate full-size cars and utility vehicles through their human controls, steering and actuating the pedals [13,14,15], though such vehicles are statically stable and require no continuous balancing. This study extends the current push toward generalist [16,17,18] manipulation and locomotion tasks to the use of two-wheeled vehicles, broadening the field of expertise of humanoid robots and demonstrating the feasibility of such a task.

### 2.2. Wheeled Control

Research on two-wheeled vehicle control can be categorized into three distinct approaches based on their control strategies. The first category comprises systems stabilized using internal gyroscopes [19,20] or counterweights [21], alongside self-balancing two-wheeled platforms of the Segway type [22,23]. These represent the majority of work in the literature and, while presenting interesting control theory challenges, they typically rely on model-based approaches that assume complete system knowledge and do not employ humanoid robots. In the second category, more advanced systems utilize steering axis actuation [24,25,26,27] to achieve stabilization. Although this approach more closely resembles real-world humanoid control, these remain specialized systems that do not broaden the field of humanoid robotics capabilities. Despite recent work demonstrating impressive agility through reinforcement learning [28], model-based approaches still dominate this category.

The third category, where humanoid robots physically operate two-wheeled vehicles, remains sparse. Small humanoid robots have been shown to balance and steer purpose-made bicycles [29], and to pedal a small bicycle while estimating the uncertain center of gravity of the robot–bicycle system to compensate for mass imbalance [30]. These systems operate vehicles built for them at their own scale, and the closest of them to the present work is reported only on a project web page rather than in the peer-reviewed literature [29]. At full size, powered two-wheelers have been operated only by custom-built mechanisms designed around a single machine and unable to perform any other task [31]. Most research in this area therefore involves small-scale, purpose-built bicycles, and to date, no humanoid robot has been documented successfully operating a full-scale, human-rideable scooter or motorcycle.

Commercial humanoid platforms have recently been shown riding two-wheeled vehicles in short promotional videos, including a 1.3 m humanoid riding a bicycle [32]. These demonstrations report no method, no evaluation protocol, and no success rate, and none has been described in the peer-reviewed literature. They also postdate the first public demonstration of the system reported here [33]. Table 1 summarizes how these systems compare with the present work.

Sim-to-real transfer of learned controllers is by now established for humanoid locomotion. Policies trained in simulation and deployed without further tuning have been shown to walk on real bipeds [34,35], to run and jump [36,37], and to carry out whole-body loco-manipulation [38], with dynamics randomization the standard mechanism for absorbing the modeling gap [11,39]. Those results transfer a policy that acts on the robot alone, and the residual gap is confined to the robot’s own dynamics. Here, the policy acts through a vehicle that is not properly modeled, coupled to the robot by a closed kinematic chain the simulator cannot represent, so the transfer must absorb the dynamics of the scooter, of the grip, and of the contact between them.

In contrast, our work extends a preliminary study conducted entirely in simulation [9] to demonstrate for the first time that a full-sized humanoid robot can operate a commercially available scooter in the real world. Moving to the physical system introduced a constraint that simulation does not impose: the steering command and the throttle are applied by the same pair of hands, so a dedicated inverse kinematics solver is required to decouple them, whereas simpler platforms separate the two by adding independent actuators. The work reported here combines simulation-driven learning, real-time inverse kinematics and sim-to-real transfer to extend the operational scope of humanoid robots to commercial vehicles operated with minimal customization.

**Table 1:** Comparison with related two-wheeled and vehicle-operating systems. *Humanoid* denotes a general-purpose anthropomorphic platform whose capabilities are not specific to a single vehicle. *Off-the-shelf system* is marked when both the robot and the vehicle are commercially available products, and *n/a* where the robot and the vehicle are a single integrated build so the distinction does not apply. *Learning* is the method used to obtain the control policy. *n/s* indicates the source does not state the property. The shaded row is an undocumented video demonstration postdating the first public demonstration of this work. A checkmark denotes that the property holds and a cross that it does not. The final row, set in bold, is the system reported here.

Work	Vehicle	Scale	Real	Humanoid	Off-the-Shelf	Learning	Sim2real
Bicycle balance control [19,20,21,24]	Bicycle	Small	✓	×	n/a	Model based	×
Self-balancing platforms [22,23]	Platform	Small	✓	×	n/a	Model based	×
Motorcycle balance control [25,26,27]	Motorcycle	Full	✓	×	n/a	Model based	×
Learned motorcycle balance [40]	Motorcycle	n/s	Sim	×	n/a	RL	×
Learned wheeled agility [28]	Wheeled	Small	✓	×	n/a	RL	✓
Humanoid vehicle driving [13,14,15]	Car, utility	Full	✓	✓	n/a	Mixed	×
Bicycle-riding humanoid [30]	Bicycle	Small	✓	✓	×	Model based	×
Bicycle-riding humanoid, unpublished [29]	Bicycle	Small	✓	✓	×	n/s	×
Full-size motorcycle rider [31]	Motorcycle	Full	✓	×	Vehicle only	Model based	×
Video demonstration [32]	Bicycle	Full	✓	✓	✓	n/s	n/s
Simulated humanoid scooter [9]	Scooter	Full	Sim	✓	✓	RL	×
**This work**	**Scooter**	**Full**	✓	✓	✓	**RL (PPO)**	✓

## 3. Simulation-to-Real Methodology

### 3.1. Simulation

To obtain a controller that stabilizes the robot while performing the desired task, a neural network is trained in simulation to map the robot’s sensory inputs to a steering command using proximal policy optimization [10]. As discussed in the introduction, the simulation environment exhibits several critical divergences from real-world conditions, including non-deformable wheels and seating material. However, the most significant limitation related to the simulator is the inability to model robot hand movements during simulation. NVIDIA’s Isaac Gym does not support closed kinematic loops without workarounds that induce numerical instabilities, which prevents accurate modeling of the robot with both hands rigidly coupled to the scooter handlebars. Isaac Gym has since been deprecated in favor of Isaac Lab, which does support closed kinematic loops. To address this constraint, two approaches were evaluated. The first trains the policy to control joint positions directly, letting the robot reach and manipulate the handlebars through collision-based interaction. The second trains the policy to output desired steering angles in simulation, which are then mapped to joint configurations by inverse kinematics during real-world deployment. The second approach was adopted as it circumvents the closed-loop kinematic constraint while maintaining effective control authority and avoiding collision-based instabilities. However, this abstraction introduces a substantial sim-to-real gap requiring careful consideration during policy transfer.

**Figure 2:**
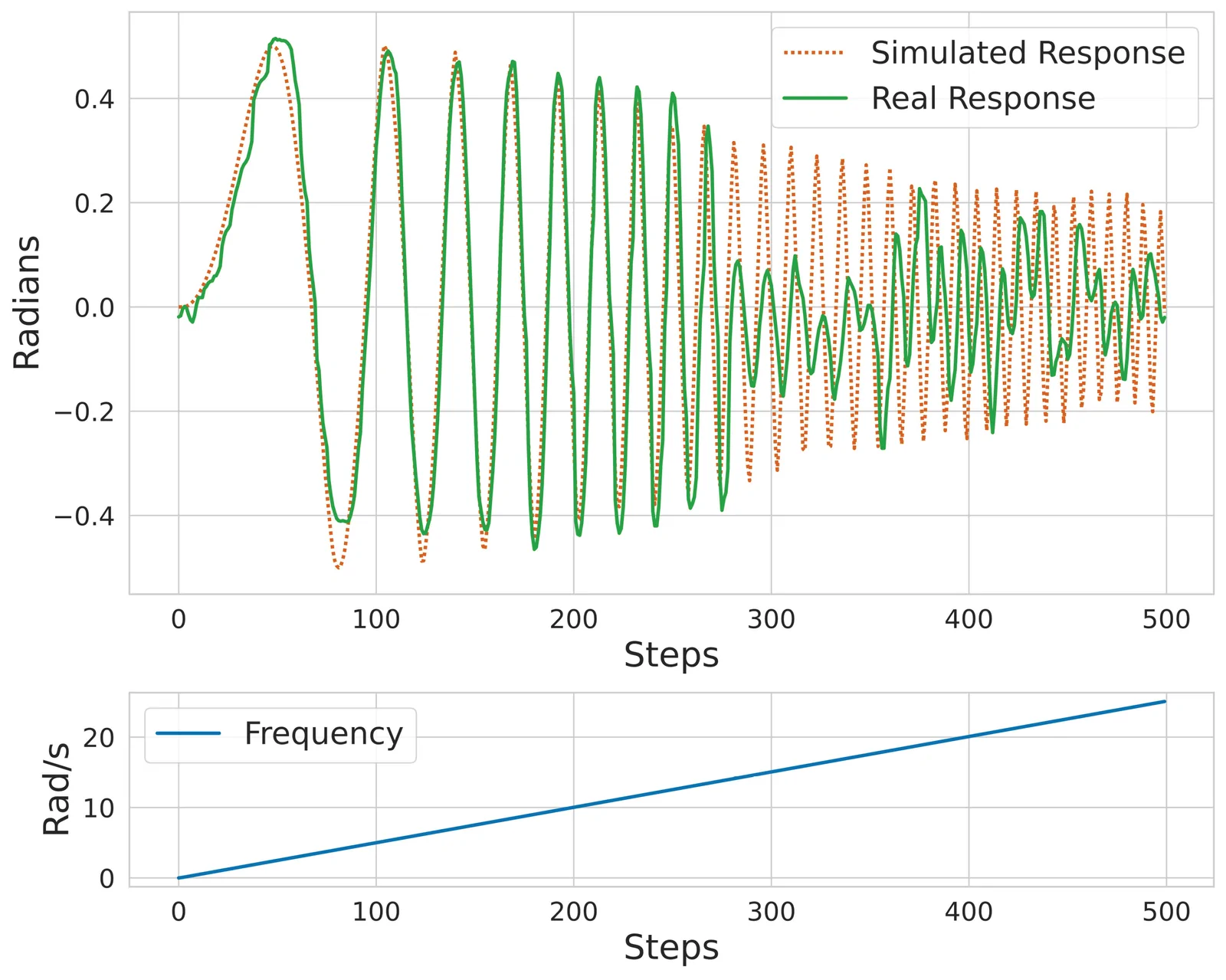
Time-domain steering response to a 0–4 Hz linear chirp: simulated system $\theta_{\mathrm{sim}}$ (orange) and real system $\theta_{\mathrm{real}}$ (green). The horizontal axis of both panels is the sample index, covering 500 samples recorded at 33.3 Hz. The (**upper**) panel shows steering angle in radians; the (**lower**) panel shows the instantaneous angular frequency of the excitation in ${\mathrm{rad}\;s}^{-1}$, with the full 0–25 ${\mathrm{rad}\;s}^{-1}$ span corresponding to the 0–4 Hz sweep. The real joint fails to follow commands above approximately 2.5 Hz.

Setting the steering axis controller gains to match real-world behavior presents a significant challenge in many scenarios. In real conditions, determining the actual gains and rotational speed of the steering joint is complex because the joint controlled in the simulation is not directly motor-controlled. As noted in the previous section, the simulation employs a fundamentally different control paradigm than the real system. In simulation, steering is achieved through direct actuation of a single virtual steering axis motor, with the robot body remaining stationary. In contrast, the real robot controls steering indirectly through coordinated motion of 14 arm-joint motors, each contributing to the net steering torque through complex coupled dynamics. This architectural mismatch complicates the tuning of simulation parameters to match real-world behavior. To address this, a simple system identification is performed by injecting a command signal *u* into both the real robot’s inverse kinematics module and the simulated scooter. We then record and compare the steering responses, shown in Figure 2 in green (real) and orange (simulated), respectively. The excitation is a linear chirp sweeping from 0 to 4 Hz over 15 s at an amplitude of ±0.5 rad, sampled at 33.3 Hz, giving a single 500-sample record; this one run is the only real-world data required for the identification. The measured response follows the command with a gain of 0.91 and a lag of 90 ms below 1 Hz, degrading to a gain of 0.51 above 3 Hz as the joint reaches its rate limit of approximately 9.4 ${\mathrm{rad}\;s}^{-1}$.

The same chirp is injected into the simulation and the proportional–derivative (PD) gains of the simulated steering joint are tuned to minimize the squared error against $\theta_{\mathrm{real}}\left(t\right)$:

(1) $$\mathrm{Error}=\sum_{t=0}^{T}{\left(\theta_{\mathrm{real}}\left(t\right)-\theta_{\mathrm{sim}}\left(t\right)\right)}^{2}$$

**Figure 3:**
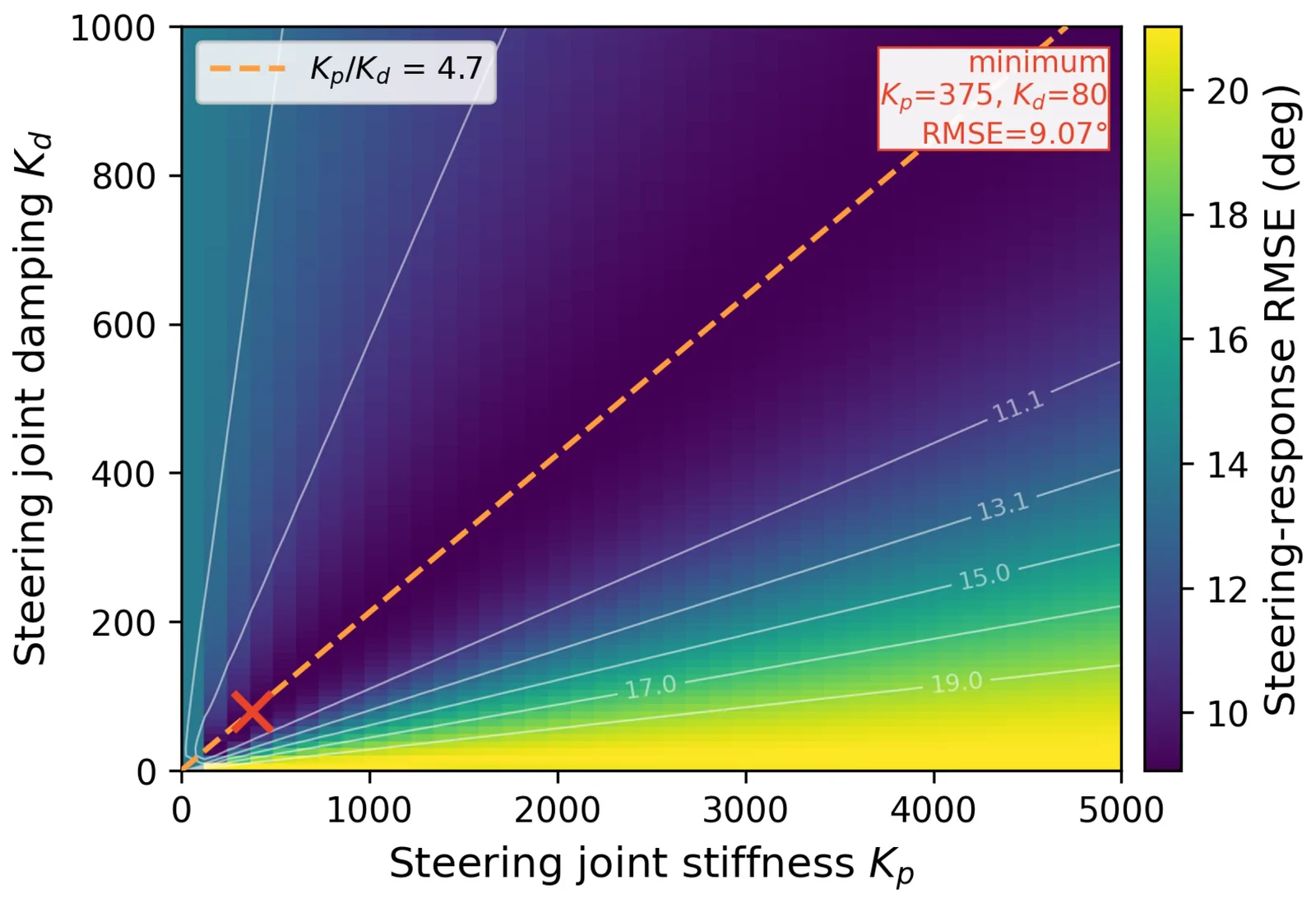
Steering-response identification error over the simulated PD gain grid. Each of the 4141 cells is one simulated environment evaluated against the same real chirp response. Error is reported as RMSE in degrees; the orange dashed line marks the ratio $K_{p}/K_{d}=4.7$ along which the fit is near-optimal, the white curves are iso-RMSE contours labeled in degrees, and the red cross marks the best fit at $K_{p}=375$, $K_{d}=80$.

The parallel-simulation capability of Isaac Gym is used to evaluate the entire gain grid at once: 4141 combinations, spanning $K_{p}\in \left[0,5000\right]$ and $K_{d}\in \left[0,1000\right]$, are simulated simultaneously, one per environment, with the scooter held fixed and every joint other than the steering joint locked. The resulting error surface is shown in Figure 3. The best fit reaches an RMSE of 9.07°, but the minimum is not isolated: all gain pairs within 0.05° of it span $K_{p}$ from 125 to 5000 and $K_{d}$ from 30 to 1000, while their ratio remains confined to $K_{p}/K_{d}=4.7\pm 0.4$. The identification therefore determines the ratio of the gains rather than their magnitude, which is why the steering damping is subsequently randomized over a wide range rather than fixed at a single identified value.

Despite being rarely seen on robotic simulated tasks and not implemented in most simulators, data augmentation is applicable to a range of control problems. Data augmentation is used in this work for faster training, as the trained policy is expected to be symmetric in the action space. Two mirrored observations are expected to generate equal and opposite actions. Only 2048 environments are simulated while 4096 are used for training; the missing half are generated by mirroring, such that an observation *o*:

(2) $$o=[\phi ,\Delta_{\psi },\omega_{\Phi },\omega_{\Psi },\theta ,v,d]$$

would give an augmented observed state $o^{\prime }$ as:

(3) $$o^{\prime }=\left[-\phi ,-\Delta_{\psi },-\omega_{\Phi },-\omega_{\Psi },-\theta ,v,d\right]$$

with $\phi ,\Delta_{\psi },\omega_{\Phi },\omega_{\Psi },\theta ,v,d$ being described in Table 2, the action *u* generated by both states should be equal but opposite:

(4) $$u\left(o^{\prime }\right)=-u\left(o\right)$$

This follows intuitively: all else being equal, falling to the right or to the left at the same angular velocity should produce equal and opposite reactions. This physical symmetry is applicable in many domains in simulators, cutting by two the computational cost of simulating new environments.

### 3.2. Domain Randomization

**Figure 4:**
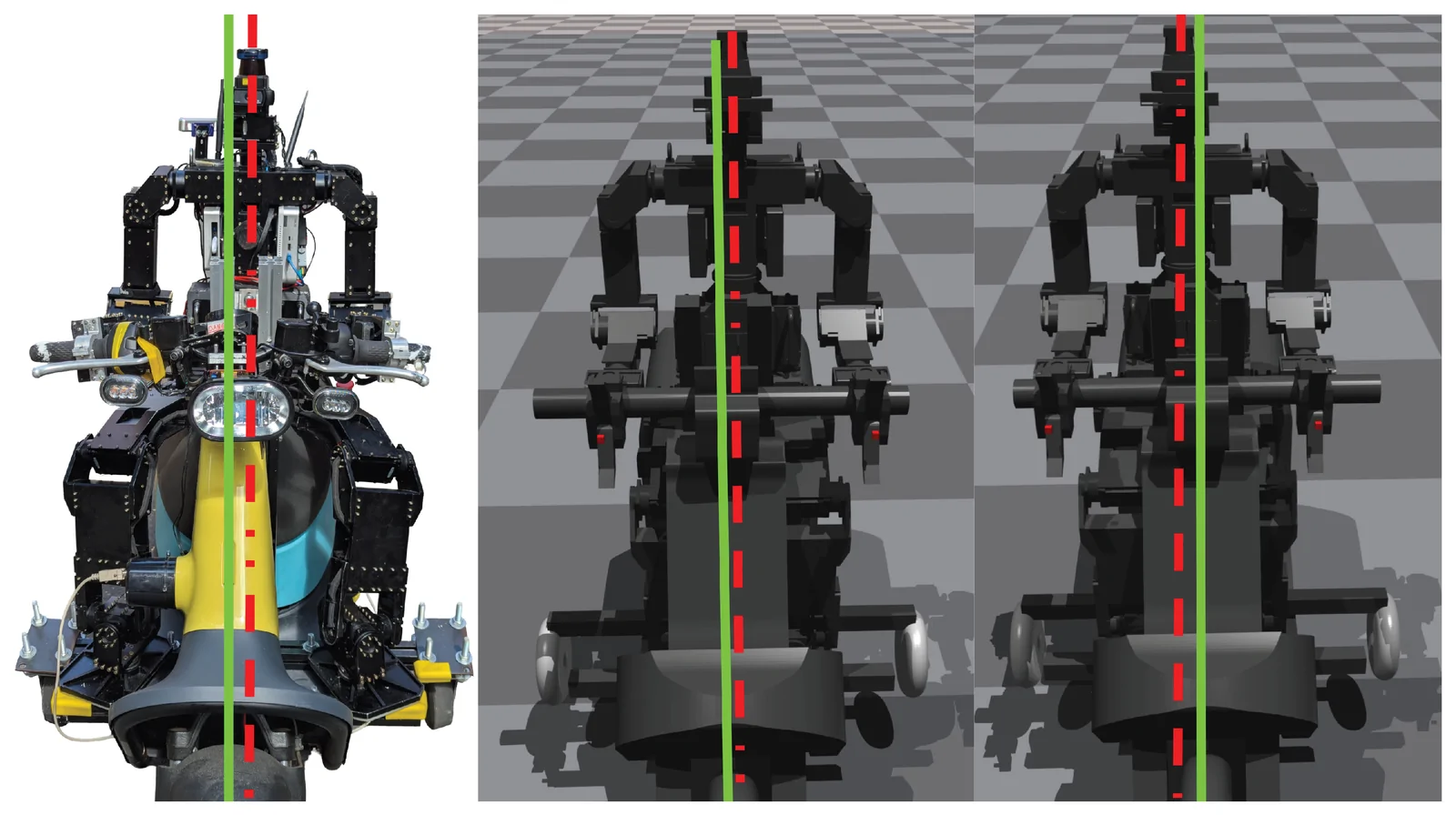
Comparison of real-life scooter seating position with simulated seating positions. The steering center is represented in green, and the seating axis in red.

After ensuring the simulated model dynamics match the real model as closely as possible, domain randomization is applied through noise and bias injection on different aspects of the simulation to allow for the learning of a more robust policy. Noise is applied following Table 3, overestimating the manufacturer datasheets. Bias is applied to model mechanical errors from the installation of the robot on the scooter, as the robot is rarely seated perfectly in the center of the scooter, as seen in Figure 4. The goal is to learn a robust policy able to control many different simulated scooters in the hope that the real scooter dynamics are included in one of those simulated scooters.

The output command of the neural network is also subject to added noise, bias, and randomized delays. The origin of each range is given in the last column of Table 3. The inertial-sensor noise ranges combine the manufacturer datasheet with the spread observed in recorded real-world data. The steering-command noise ranges follow from the measured precision of the inverse-kinematics solution, and the forward-velocity noise and bias from the spread of the SLAM velocity estimate. The steering damping follows from the identification procedure of Section 3. The remaining ranges are engineering estimates rather than measurements. The mounting-alignment bias, the seating positions and the spawn attitude were estimated and then widened beyond the expected values, so that the true parameter falls inside the sampled interval. The command delay was estimated from the latency of the control stack between the host computer and the motor controllers. The gravity, rigid-body mass and external-push ranges are broad margins around their nominal values, chosen without a dedicated measurement campaign. We report this distinction explicitly rather than presenting all ranges as measured. Percentages are expressed relative to an explicit reference: 100% steering damping corresponds to the nominal value of 200 used in simulation, which lies on the ratio $K_{p}/K_{d}=4.7$ identified in Figure 3, and 100% rigid-body mass corresponds to the nominal mass of each body in the robot–scooter model. Uniform ranges are bounded by construction; the Gaussian observation noises are applied to sensor readings and are not clipped, since their standard deviations are small relative to the observation ranges reported in Table 2 and clipping would misrepresent the sensor behavior being modeled. Every simulated environment running in parallel has different biases, and every bias is changed during every reset of the environment, helping generalization.

Different simulation parameters are also randomized between every environment, such as the delay in the command, the damping of the steering axis, and other parameters shown in Table 3. Additionally, an external lateral push to the head is applied and changed every 10 s to simulate external perturbations.

**Figure 5:**
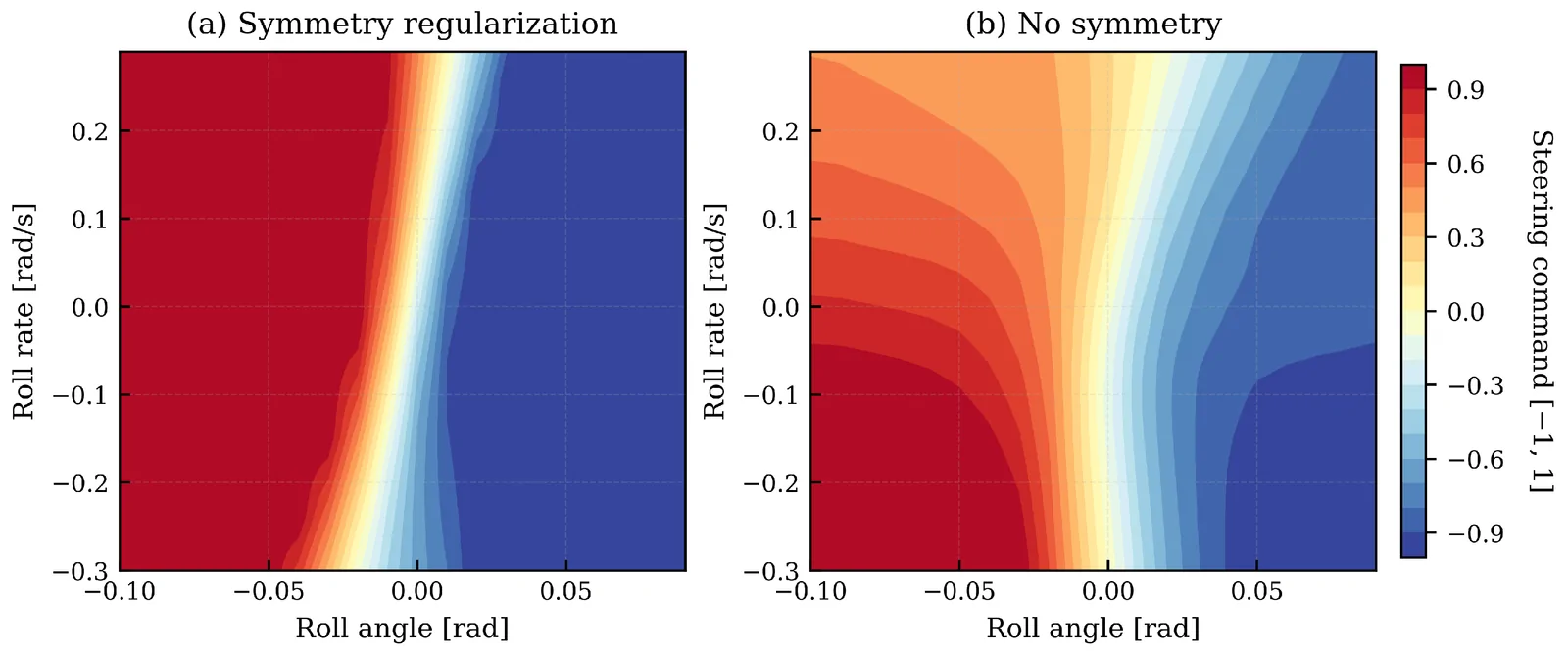
(**a**) Symmetry regularization compared with (**b**) vanilla PPO policy response after 100 epochs. Both panels show the policy’s steering command over the roll angle/roll rate plane, on a common color scale.

Symmetry is exploited in two distinct ways. The first, described in Section 3, is data augmentation: mirrored observations supply half of the training batch, halving the simulation cost per sample. The second is reward regularization through the term $r_{\mathrm{symmetry}}$, which penalizes asymmetric responses to mirrored observations and thereby encourages the policy to acquire the physical symmetry of the system earlier in training. Figure 5 shows the response of the neural network output given different roll angles and angular velocity at the same training step. The symmetry reward helps the policy quickly learn the symmetry of the physical system it embodies. This additional reward, however, does not influence final performance as the policy eventually learns the symmetry by simply interacting with the environment.

### 3.3. Training

Sensory inputs used to define a full system observation are shown in Table 2. A state history consisting of the last 20 observations (0.2 s) is fed to the network, which outputs steering angles that are then forwarded to the simulation to control the steering axis directly. The window was chosen to exceed the largest command delay applied during domain randomization (0.15 s, Table 3). A delayed steering command therefore still lies within the observation window when it takes effect, so the policy can associate its past actions with their delayed consequences. For safety considerations, the neural network is not trained to control the throttle of the scooter; instead, the target speed is set by the user and the robot must adapt its control to that speed.

The reward function *R* used to train the policy is a weighted sum of several components refined to optimize different aspects of the system’s behavior. The components are described below:

(5) $$\begin{array}{cc} r_{\psi } & =1-{\Delta_{\psi }}^{2}\\ r_{\phi } & =1-\phi^{2}\\ r_{\omega } & =1-{\omega_{\Phi }}^{2}\\ r_{\mathrm{actionpenalty}} & =1-\left(u{\left(o\right)}^{2}\;\cdot \;w_{\phi }\;\cdot \;w_{\omega }\right)\\ r_{\mathrm{smoothness}} & =1-{\left(\frac{\partial }{\partial t}u\left(o\right)\right)}^{2}\\ r_{\mathrm{symmetry}} & =1-{\left(\frac{u\left(o\right)+u\left(o^{\prime }\right)}{2}\right)}^{2} \end{array}$$

where u(o) is the action taken by the policy network given the previously described observation and $w_{\phi }$ and $w_{\omega }$ being weight parameters to avoid penalization of the actions when the robot is falling:

(6) $$\begin{array}{cc} w_{\phi } & =1-\tanh(50\;\cdot \;\phi^{2}),\\ w_{\omega } & =1-\tanh(50\;\cdot \;\omega_{\Phi }^{2}), \end{array}$$

Finally the reward function is defined as:

(7) $$\begin{array}{cc} R=r_{\psi } & +r_{\phi }\;\cdot \;0.1\\ \; & +r_{\omega }\;\cdot \;0.35+r_{\mathrm{actionpenalty}}\;\cdot \;2.0\\ \; & +r_{\mathrm{smoothness}}\;\cdot \;0.2+r_{\mathrm{symmetry}}\;\cdot \;3.0 \end{array}$$

The reward weights were selected empirically during development rather than by systematic search. The roll, roll-rate, action-penalty, and smoothness weights (0.1, 0.35, 2.0, and 0.2, respectively) were fixed early and retained unchanged thereafter, as was the symmetry weight of 3.0 once that term was introduced. The yaw-tracking weight required the most iterations, since it governs the trade-off between path tracking and dynamic stability. Substantially lower values yield a policy that balances reliably but under-steers in turns; higher values recover tracking at the cost of stability margin during tight maneuvers. The value of 1.0 used for the reported policy was selected on this basis.

### 3.4. Network Parametrization

Expanding the randomization domain increases the observation space covered during training, which can help ensure that real-world scenarios are adequately represented; however, this expansion introduces trade-offs: it slows down training convergence [41], increases sample complexity, and often leads to suboptimal performance [11]. A careful analysis of the real system can help minimize the domain expansion. However, certain parameters of real-world systems can be difficult to determine or outright impossible to estimate in advance, necessitating a large randomization domain to ensure that the true real-world values are not missed. For example, estimating command delay in complex systems can be particularly challenging without a completed system for direct measurement. To address this issue, some observations can serve as proxies for unknown system parameters, enabling the neural network to incorporate a form of parametrization that can be adjusted during real-world testing. The *d* observation seen in Table 2 is used to inform the neural network of the current command delay, which is randomized in simulation and then can be tuned during real-life operations as in a human-guided online system identification [42]. This approach reduces the need for retraining the policy repeatedly with varying parameters, as the network can adapt its control behavior in real time based on these user-provided observations. This type of user-controlled observation has demonstrated significant benefits during testing and has a negligible impact on the training speed. In practice, *d* is calibrated once rather than tuned continuously. Its value was selected empirically before the reported experiments using straight-line driving runs, in which the parameter was varied and the resulting change in tracking and stability observed. The selected value was then held fixed for all reported experiments and was not adjusted between runs or between scenarios: the same calibration was used for the turning, speed-bump, passenger-carrying, and license-test experiments. The parameter therefore acts as a one-off calibration of the platform rather than a control input requiring continuous operator attention. The system is nevertheless not self-adaptive in this respect, and online estimation of the delay is left to future work. The network is not required to learn a universal control policy robust to all potential variations; instead, it is provided with information about the current conditions and applies the control policy suited to the specific case. This reduces the burden on the policy to generalize across the full range of randomization while improving real-world adaptability and performance. In the future, we plan to use model-based methods to estimate those parameters so the robot can self-tune while driving.

The complete configuration used to train the reported policy is given in Table 4, and the full training code, task and training configuration files, domain-randomization parameters, and evaluation scripts are released with this paper.

Training was terminated on a wall-clock budget rather than at a fixed number of environment steps. The reported policy, and each configuration in the ablation study of Section 5.3, corresponds to a single training run with a single random seed; the implications of this for the interpretation of the ablation results are discussed in Section 6.

### 3.5. Seating Identification and Inverse Kinematics

The robot is positioned on the scooter, with its arms securely connected to the scooter’s handles by an operator. To maintain the realism of the experiment, no specialized alignment systems are employed on the seat or handles. Consequently, each time the robot is seated, the relative positions of its hands and base with respect to the scooter are initially unknown. However, once the robot is fixed to the seat and its hands are clamped to the handles, precise knowledge of its positioning becomes critical for generating accurate hand trajectories and preventing excessive motor torques if the hand were to deviate from the virtual steering circle. To achieve this, an initialization procedure determining the precise position of the robot and its grippers compared to the center of steering is required. Without this step, attempts at controlling the robot would result in movements exceeding the torque limitations of the actuator.

**Figure 6:**
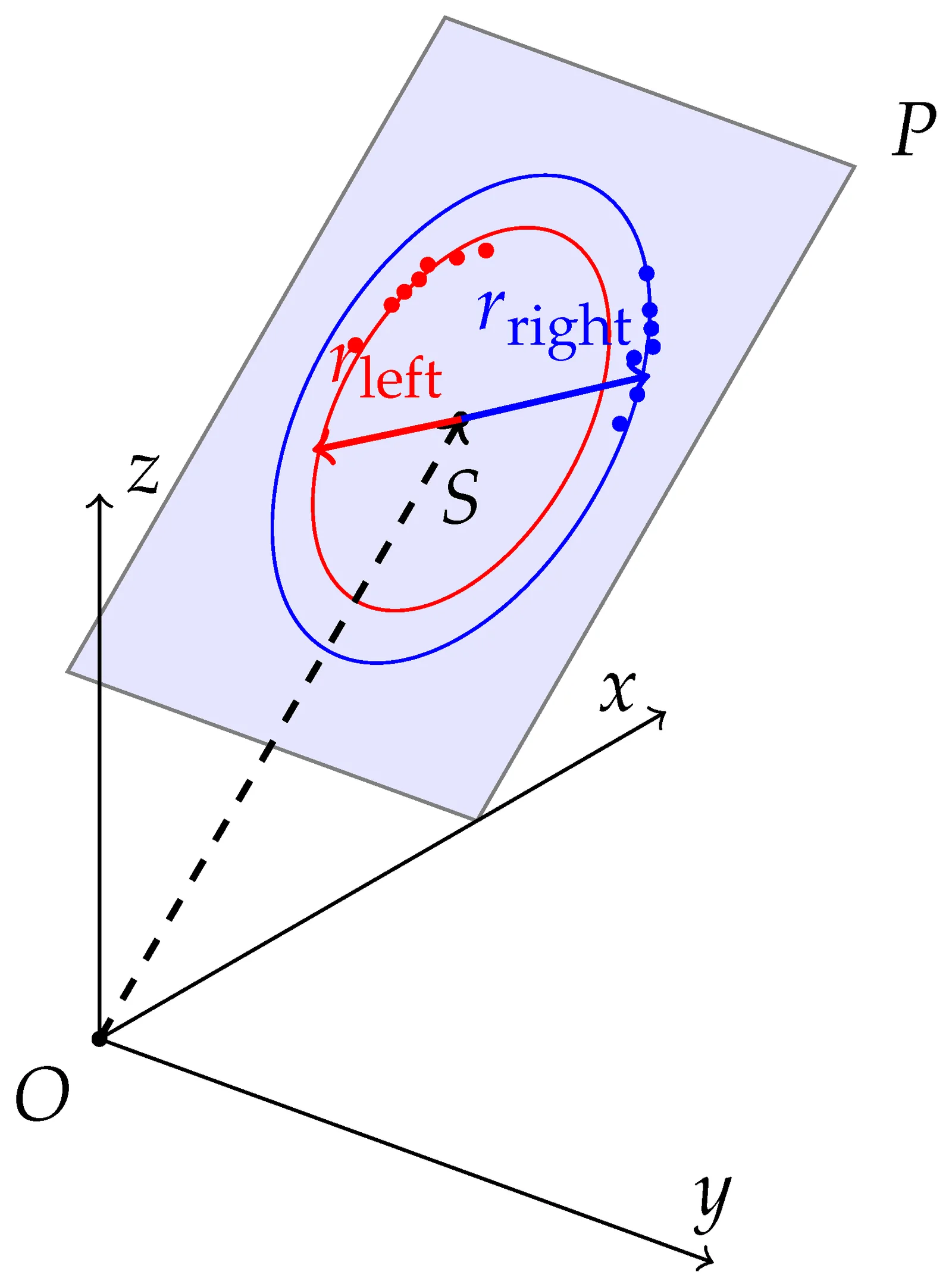
Geometry of the seating identification. The robot base frame is at *O*, with *x* forward and *z* upward. The measured hand positions of the two arms, shown as red and blue points, are fitted by a common plane; within that plane each hand follows a circle about the steering center S with its own radius $r_{\mathrm{left}}$ or $r_{\mathrm{right}}$.

All positions and orientations in this section are expressed in the robot’s base frame, shown in Figure 6, with the *x* axis pointing forward, the *z* axis upward, and the *y* axis completing a right-handed frame. The plane parameters, the steering center S and the hand trajectories are resolved in that frame. The signs in Equation (15) follow from this convention: the normal is taken with C>0, pointing upward; $P_{\phi }$ is a rotation about *y* taken as +arcsin(A/∥n∥) and $P_{\theta }$ a rotation about *x* taken as −arctan2(B,C), so that $M_{P}$ maps the *z* axis onto n/∥n∥ and a level steering plane gives the identity.

Assume that for each arm *a* (with a∈{left,right}), we collect a set Q of *N* joint configurations q:

(8) $$Q_{a}=\left\{\;q_{a,i}\in R^{7}|i=0,\ldots ,N-1\;\right\},$$

which are mapped via the forward kinematics function FK to obtain the 3D hand positions $X_{a}$:

(9) $$X_{a}=\left\{\;x_{a,i}={\mathrm{FK}}_{a}\left(q_{a,i}\right)\in R^{3}|i=0,\ldots ,N-1\;\right\}.$$

The combined left-arm and right-arm point set is noted X without subscript:

(10) $$X=X_{\mathrm{left}}\cup X_{\mathrm{right}}.$$

We assume that the hand positions lie on a plane *P* following the equation:

(11) $$n\;\cdot \;x-D=0,\;\mathrm{with}\;n={\left(A,B,C\right)}^{⊤},\;C>0,$$

where the plane parameters A,B,C,D can be obtained by solving a constrained least-squares problem:

(12) $$\min_{n,\;D}\sum_{x_{i}\in X}{\left(n\;\cdot \;x_{i}-D\right)}^{2},\;\mathrm{subject}\;\mathrm{to}\;∥n∥=1$$

Let $S\in R^{3}$ be the center of the steering rotation and let the hand trajectories be circles with radius $r_{a}$ (with again a∈{left,right}) centered around S. Since the steering plane *P* is not coplanar with the xy-origin plane, we constrain the *z*-coordinate of S by projecting S onto *P* as follows:

(13) $$S_{z}=\frac{AS_{x}+BS_{y}-D}{-C}$$

Then, the optimization for the hand parameters (i.e., $S_{xy}={\left(S_{x},S_{y}\right)}^{⊤}$ and $r_{a}$) is formulated as

(14) $$\min_{S_{xy},\;r_{\mathrm{left}},\;r_{\mathrm{right}}}\sum_{a\in \{\mathrm{left},\mathrm{right}\}}\sum_{i=0}^{N-1}{\left(∥x_{a,i}-S∥-r_{a}\right)}^{2}$$

We define a single rotation matrix $M_{P}$ that aligns the steering plane *P* with the robot’s base frame. One approach is to compute the Euler angles from the normal vector n. We define the plane tilt and roll angle as:

(15) $$P_{\theta }=-\arctan2\left(B,\;C\right),\;P_{\phi }=\arcsin\left(\frac{A}{∥n∥}\right)$$

and define the respective rotation matrices as:

(16) $$M_{\theta }=\left[\begin{array}{ccc} 1 & 0 & 0\\ 0 & \cos P_{\theta } & -\sin P_{\theta }\\ 0 & \sin P_{\theta } & \cos P_{\theta } \end{array}\right],\;M_{\phi }=\left[\begin{array}{ccc} \cos P_{\phi } & 0 & \sin P_{\phi }\\ 0 & 1 & 0\\ -\sin P_{\phi } & 0 & \cos P_{\phi } \end{array}\right]$$

Then, the overall rotation is defined as:

(17) $$M_{P}=M_{\theta }\;M_{\phi }.$$

With these angles $M_{P}\;{\left(0,0,1\right)}^{⊤}={\left(\sin P_{\phi },\;-\sin P_{\theta }\cos P_{\phi },\;\cos P_{\theta }\cos P_{\phi }\right)}^{⊤}=n/∥n∥$, so the flat circle of Equation (18) is carried exactly into the fitted plane.

### 3.6. Mapping Steering to Hand Trajectories

For each arm *a*, we parameterize the hand trajectory by an angle φ (which is mapped from the steering command) and an offset $\delta_{a}$ (with, for example, $\delta_{\mathrm{right}}=-\mathrm{offset}$ and $\delta_{\mathrm{left}}=+\mathrm{offset}$). Define the pre-rotation hand position on the circle by

(18) $$X_{a}^{\prime }\left(\varphi \right)=\left[\begin{array}{c} r_{a}\cos\left(\varphi +\delta_{a}\right)+S_{x}\\ r_{a}\sin\left(\varphi +\delta_{a}\right)+S_{y}\\ S_{z} \end{array}\right].$$

Then, the final hand position $X_{a}$ of the hand a∈{left,right} in the base frame is given by:

(19) $$X_{a}\left(\varphi \right)=M_{P}\;\left(X_{a}^{\prime }\left(\varphi \right)-S\right)+S$$

Similarly, the tangent vector (before rotation) is

(20) Ta′(φ)=−rasinφ+δa−racosφ+δa0

and its rotated version:

(21) $$T_{a}\left(\varphi \right)=M_{P}\;T_{a}^{\prime }\left(\varphi \right).$$

From the rotated tangent vector $T_{a}\left(\varphi \right)={\left(T_{x,a},T_{y,a},T_{z,a}\right)}^{⊤}$, we compute the pitch and yaw of the hand. A single direction vector fixes only two rotational degrees of freedom; the remaining rotation about the tangent is set by the grip rather than by $T_{a}$, and the hand roll $\phi_{a}$ is therefore held at a fixed value for each arm, $\phi_{\mathrm{left}}=-87.1$° and $\phi_{\mathrm{right}}=87.1$°:

(22) $$\theta_{a}=\arctan2\;\left(-T_{x,a},\;\sqrt{T_{y,a}^{2}+T_{z,a}^{2}}\right)+\theta_{0}-1_{\left\{a=\mathrm{right}\right\}}\;k_{t}\;\mathrm{Throttle}$$

(23) $$\psi_{a}=\arctan2\;\left(T_{y,a},\;T_{x,a}\right),$$

where $\theta_{0}=0.2\;\mathrm{rad}$ is a constant pitch offset applied to both hands, Throttle∈[0,1] is the normalized policy output and $k_{t}=0.7\;\mathrm{rad}$ is the angle through which the right hand rotates the grip between a closed and a fully open throttle. The three angles are applied to the hand as roll about *x*, then pitch about *y*, then yaw about *z*, so that $R_{a}=R_{z}\left(\psi_{a}\right)\;R_{y}\left(\theta_{a}\right)\;R_{x}\left(\phi_{a}\right)$.

Once the target hand pose positions and rotations are defined, we can solve the second problem of finding the correct joint configuration to reach the desired pose. This classical inverse kinematics problem is divided into two parallel computations, one for each hand, and is considered finished when both solutions are returned. The optimization is solved by the limited-memory BFGS-B C++ implementation [43], solving both arms well within the 100 Hz control period. Table 5 reports the measured solve time over 10,000 calls: the median solve takes 91 μs from the recorded start pose and 21 μs when seeded with the previous solution, against a control period of 10 ms. Hand orientations are represented as roll, pitch, and yaw applied in the order *x*, *y*, *z*, and are recovered from the corresponding rotation matrix with the two-argument arctangent, the pitch being taken on the principal branch. The arm has no kinematic singularity within the range of poses reachable for any steering and throttle combination. Self-collision is prevented by the bounds on the shoulder roll, which is held at least 68° away from the torso on each arm, so the arms remain clear of the body throughout. Joint limits are not applied after the fact but enter the optimization directly as box constraints on the L-BFGS-B solve, and a solution that leaves those bounds, or a solve that fails to converge, is rejected rather than executed. It is worth noting that employing a neural network to directly output all joint positions or torques is a feasible and widely used approach in certain control tasks, such as walking. However, this approach was deliberately avoided in this work. The implemented inverse kinematics addresses both direct joint control issues by ensuring the generated solutions maintain the robot’s hands securely on the steering bars. Specifically, the control outputs Steering∈[−1,1] and Throttle∈[0,1], are mapped to joint positions via the inverse kinematics module, ensuring proper joint control while mitigating safety and closed-loop kinematic challenges.

## 4. Experimental Setup

### 4.1. System Architecture

The experimental setup involves the Thormang3 (ROBOTIS Co., Ltd., Seoul, Republic of Korea) humanoid robot [44] operating a commercially available Gogoro Viva scooter (Gogoro Inc., Taoyuan, Taiwan) [45]. The robot has no roll degree of freedom to control its upper body, so it cannot lean into turns to shift its center of mass as a human rider would; all balancing and cornering authority must be exerted through steering control alone. The robot–scooter system has been augmented with minimal modifications to keep a maximum of realism while enhancing safety and ease of use during testing. Significant preliminary effort was dedicated to ensuring the safety of the experiment. Those hardware modifications include the following:

- A set of end effectors enabling easier grip of the handles.
- A safety frame allowing the robot to stay on the scooter in case of an accident and preventing the robot from flipping the scooter in case of control loss.
- A remote-controlled 34 Nm servomotor connected to the scooter’s hydraulic brake, independent of the robot–scooter system, allowing the operator to bring the scooter to a total stop in case of an emergency.
- A control board connected to the scooter motor control bus, allowing the maximum speed of the scooter to be manually capped by the turn of a potentiometer. The device is also used as a physical motor cutoff.

No incident, fall or injury has occurred at any point in this work, from the experiments reported in this paper up to the present day, thanks to the strict safety procedures described above.

The scooter–robot system is launched by hand for the first 2 to 3 m until the minimal stabilization speed of 0.3 ${m\;s}^{-1}$ is reached in all experimental setups. In the same vein, the robot–scooter system is stopped using the emergency brake and the robot does not use its legs to perform a controlled stop.

### 4.2. Success Criterion

A run is scored as a success when the robot–scooter system completes the full maneuver under two conditions: the stabilization wheels of the safety frame never contact the ground, and the system never leaves the roadway. The first condition is a direct measure of balance. The stabilization wheels touch down at a roll angle of approximately 22°, which coincides with the 0.38 rad roll limit used to terminate episodes in simulation, so any contact marks a loss of balance from which the policy can no longer recover. Consequently, the stabilization wheels do not contact the ground during any successful trial and play no mechanical role in the balance control reported here. The second condition is scored by contact with the sidewalk or the road boundary. The operator applies the remote emergency brake when either condition is met or is imminent; a run terminated by the emergency brake is therefore always scored as a failure. Beyond the manual launch over the first 2 to 3 m, the operator makes no contact with the robot or the scooter, and every reported run is completed hands-off.

This criterion applies to all maneuvers in Table 6 and to the speed-bump and passenger-carrying scenarios in Table 7. The license-test corridor is scored by the rules of the standardized test itself, which add two requirements to the above: the system must remain inside the 0.4 m corridor, and must take no less than 7 s to traverse its 15 m length. Violating either scores the run as a failure.

## 5. Results

### 5.1. Standard Scenarios

To comprehensively evaluate the system’s capabilities, real-world experiments across multiple scenarios of increasing complexity are conducted. The number of trials differs between maneuvers and is reported for each in Table 6, where success rates are given as the number of successful trials over the number attempted. During tests the speed varies across the operating envelope and averages 1.5 ${m\;s}^{-1}$, with a maximum controllable speed at 3 ${m\;s}^{-1}$ due to safety concerns and training envelope. All experiments are conducted on real-world road conditions, consisting primarily of asphalt surfaces with notable irregularities and a high crown of 3 to 6%, which far exceeds typical road crowns (usually 1–3%). The test route includes sections of aggregate surface and tile paving, introducing additional variability in surface friction and texture that further challenged the system’s adaptability. The longest straight section used during testing is approximately 150 m.

**Figure 7:**
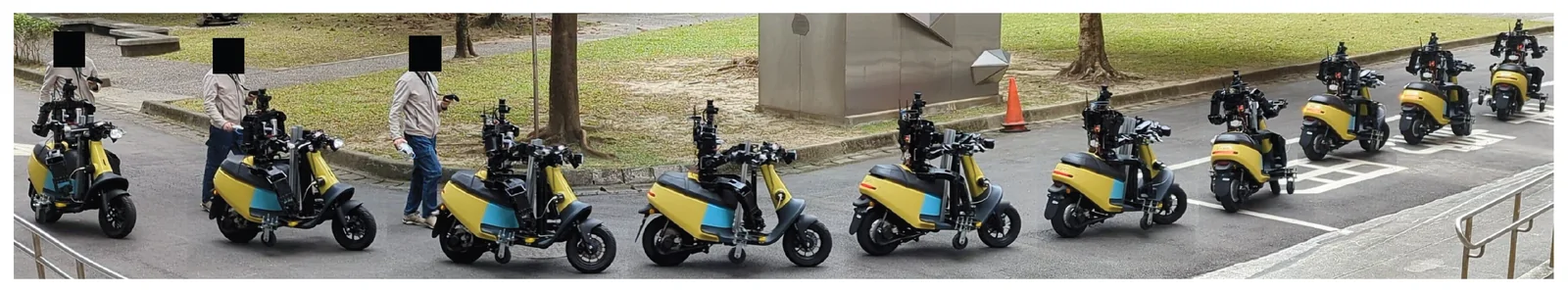
Motion sequence of the Robot–Scooter system completing a 90-degree turn.

Tests are divided into two sections: standard driving and challenging scenarios. During standard driving performance tests, the system is evaluated on four fundamental maneuvers: straight-line driving, light trajectory adjustment turns under 50°, turning operations above 90°, as seen in Figure 7, and roundabouts.

**Figure 8:**
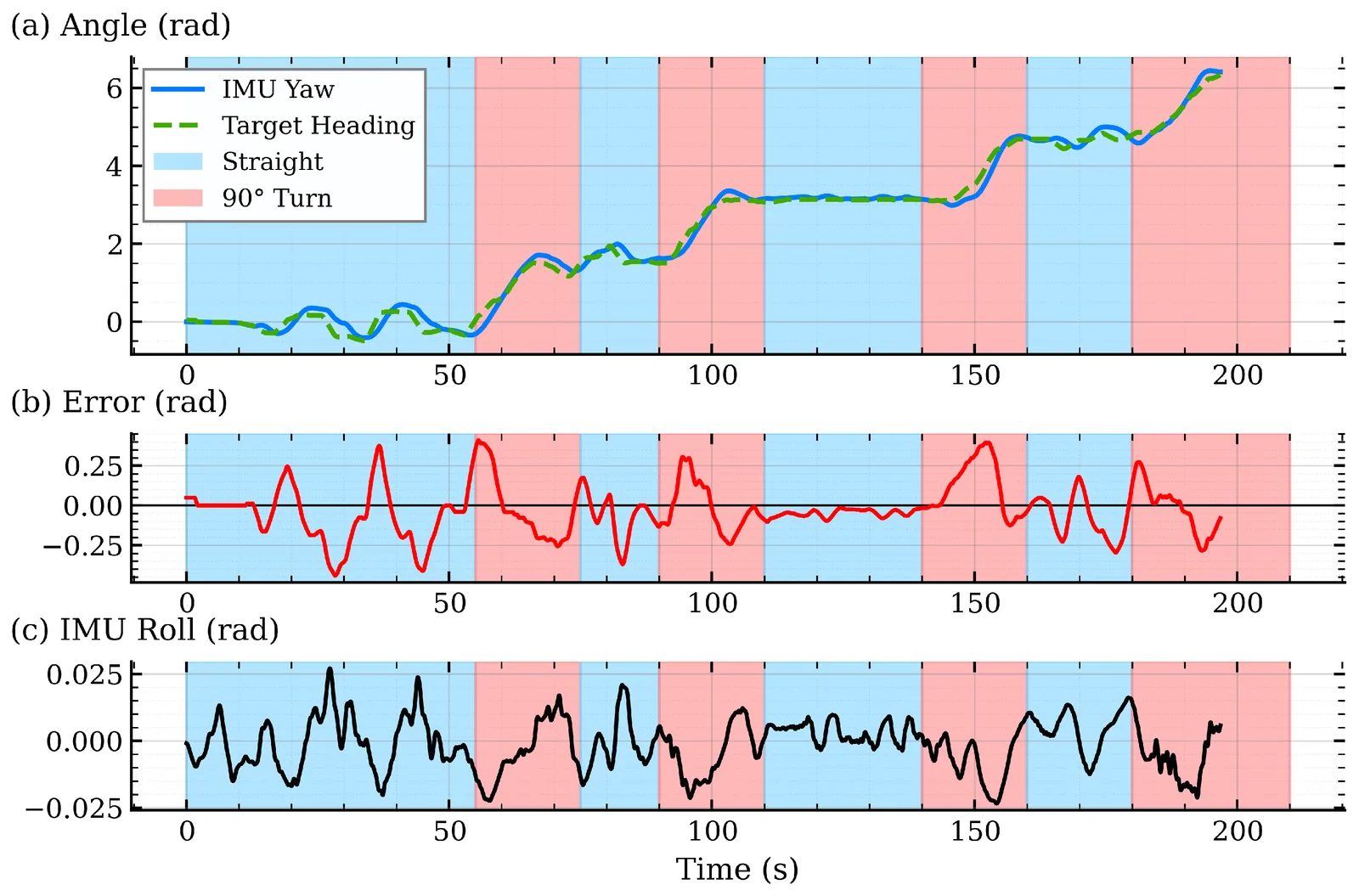
System yaw tracking performance: (**a**) measured yaw angle and commanded target heading from remote control, (**b**) tracking error, drawn in red with the black horizontal line marking zero error, and (**c**) roll angle, drawn in black. Blue and pink shaded regions denote straight and 90° turn segments. The figure shows a single instrumented run, chosen because it displays the tracking behavior clearly.

Table 6 presents the success rates of the standard driving scenarios, while straight-line driving is very reliable over time, a tighter turning radius poses a greater challenge to the control policy, due to the robot’s inability to move its body and its reliance on steering control alone. Additionally, the system exhibits a tendency to prioritize dynamic stability over aggressive trajectory tracking when commanded to execute sharp turns, as evidenced by lower success rates for sharper turns. Figure 8 shows the tracking error in the light turn scenario and the 90-degree turn scenario during free driving exercises. Tracking error is higher during sharper turns.

### 5.2. Challenging Scenarios

**Figure 9:**
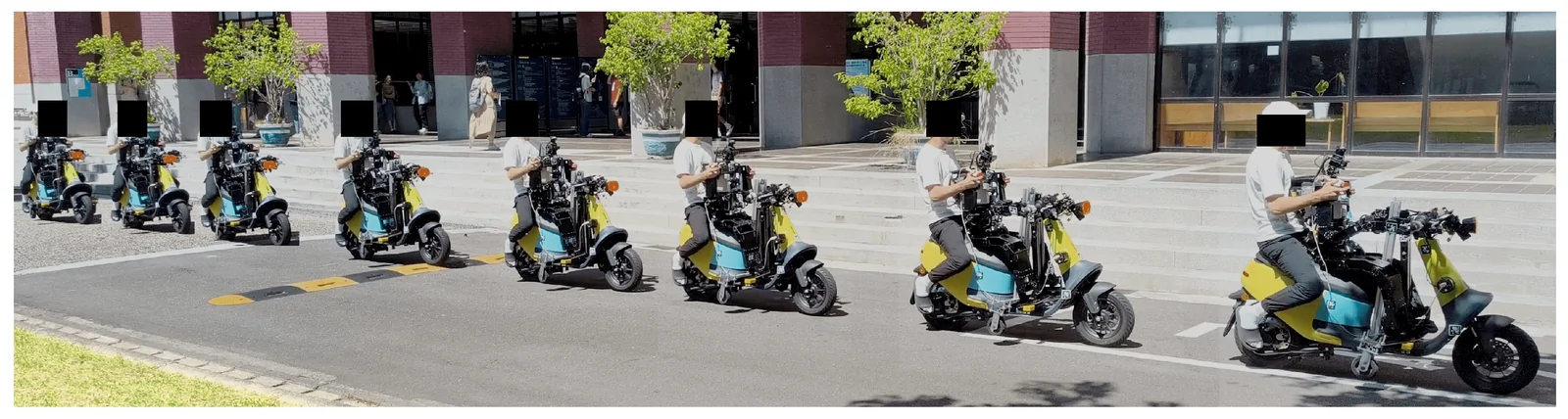
Robot–Scooter system riding over a bumper while carrying a human passenger (2 ${m\;s}^{-1}$).

**Figure 10:**
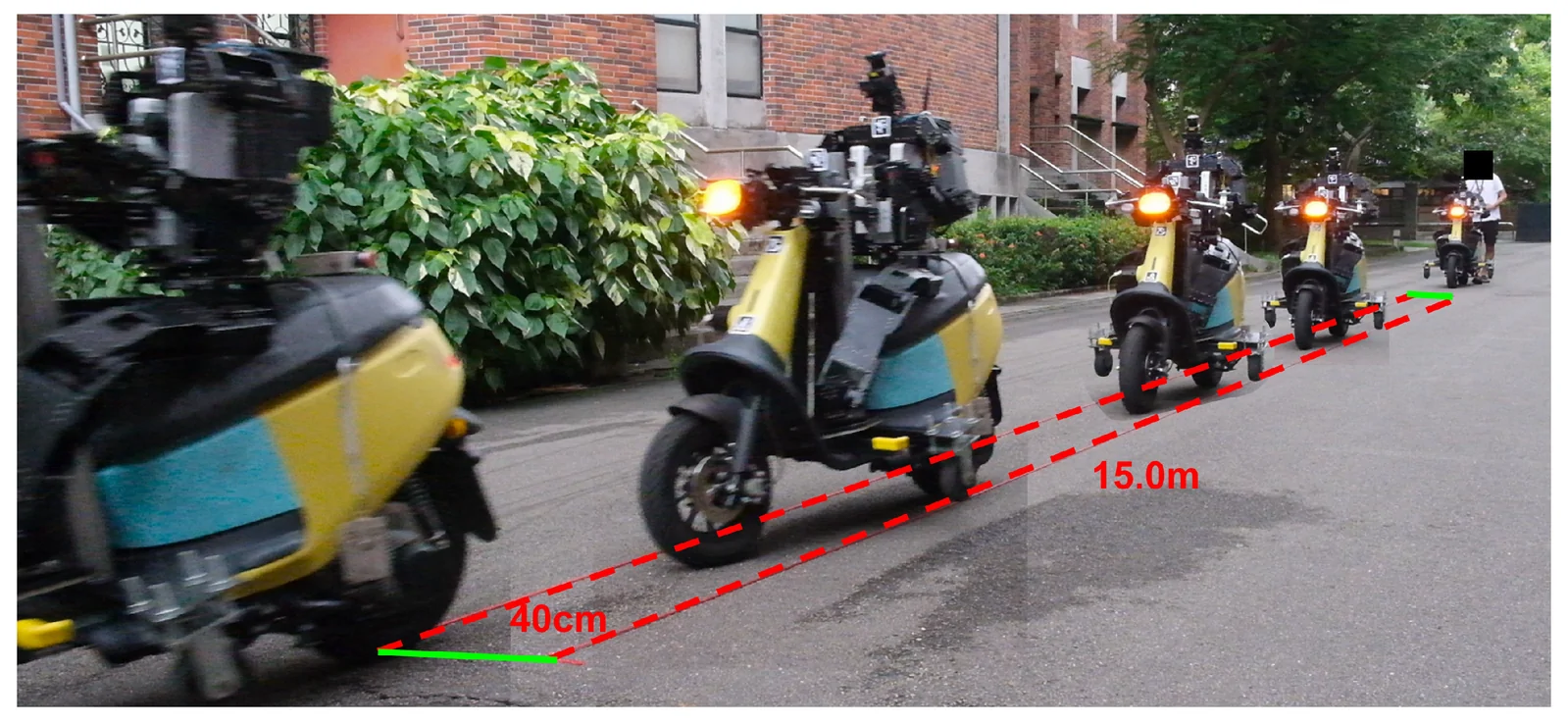
Robot–Scooter passing the Taiwanese scooter license challenge.

Challenging scenarios assess the system’s robustness beyond standard operation through three demanding tasks. The first is obstacle traversal, in which the robot crosses a 60 mm speed bump while maintaining balance. The second is dynamic load handling, with a human passenger of approximately 70 kg seated on the rear. The third is precision navigation through the Taiwanese motorcycle license test’s narrow corridor, a passage 15 m long and 0.4 m wide that must be traversed in no less than 7 s. Figure 1, Figure 9 and Figure 10 show the three tasks being performed. Performance is reported quantitatively in Table 7, which summarizes the success rates across all scenarios. The Taiwanese license test remains challenging for the robot, at a success rate of 0.33, because the path-tracking response prioritizes the stability of the system. The robot’s longest successful traverse of the corridor was 12 s, obtained with the stabilization wheels and the speed limiter of Section 4 fitted. An instructor at a scooter driving school stated in discussion that he can hold the corridor for approximately 11 s on an unmodified scooter. Speed-bump traversal and passenger carrying are less affected, with success rates of 0.88 and 0.73, respectively, against a baseline of 0.88 for standard free driving, an unstructured route combining straight sections with occasional light turns rather than the straight-line maneuver of Table 6. The passenger result was obtained without retraining and across varied seating positions.

**Figure 11:**
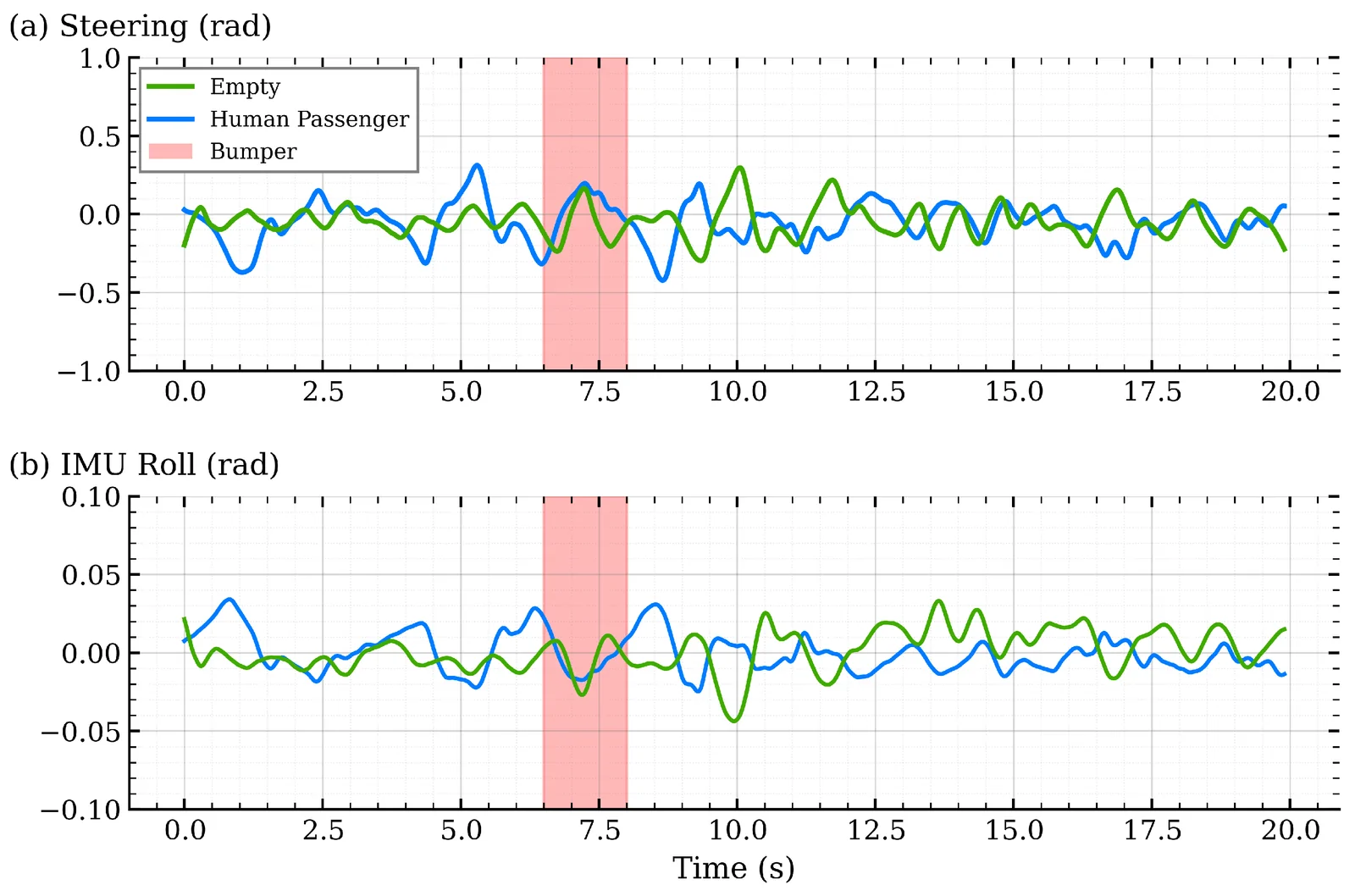
Comparison of driving sequences with the robot scooter system carrying a human (blue) and without (green). (**a**) Steering angle and (**b**) roll angle; the shaded band marks the speed-bump traversal. Each condition is shown by one recorded run, neither of which was selected for quality.

To evaluate the system’s robustness under significant dynamic loads, we conducted tests with a human passenger (approximately 70 kg) seated on the rear of the scooter as seen in Figure 9. All trials involving a human passenger followed the same procedure. The passenger was an author of this paper rather than an independent volunteer, so no third party was exposed to any risk related to the robot’s driving skills. Figure 11 compares passenger-carrying and robot-only operation over the same test section. Panel (**a**) shows the steering angle and panel (**b**) the roll angle, for both conditions. Despite the substantial mass addition and shift in center of gravity, the controller keeps the roll angle within the same range in both conditions, and the steering corrections in panel (**a**) follow similar patterns. The seating-position and mass randomization applied during training is a plausible explanation for this robustness, and removing weight randomization does reduce the free-driving success rate from 0.88 to 0.71 in the ablation study of Section 5.3. The comparison of Figure 11 is illustrative rather than quantitative: the comparison between the two conditions rests on the success rates of Table 7, 14/16 without a passenger and 8/11 with one, whose confidence intervals overlap. Panel (**b**) shows the robot holding its roll angle within 0.1 rad while traversing a speed bump at 2 ${m\;s}^{-1}$, marked by the shaded band.

### 5.3. Ablation Study

**Figure 12:**
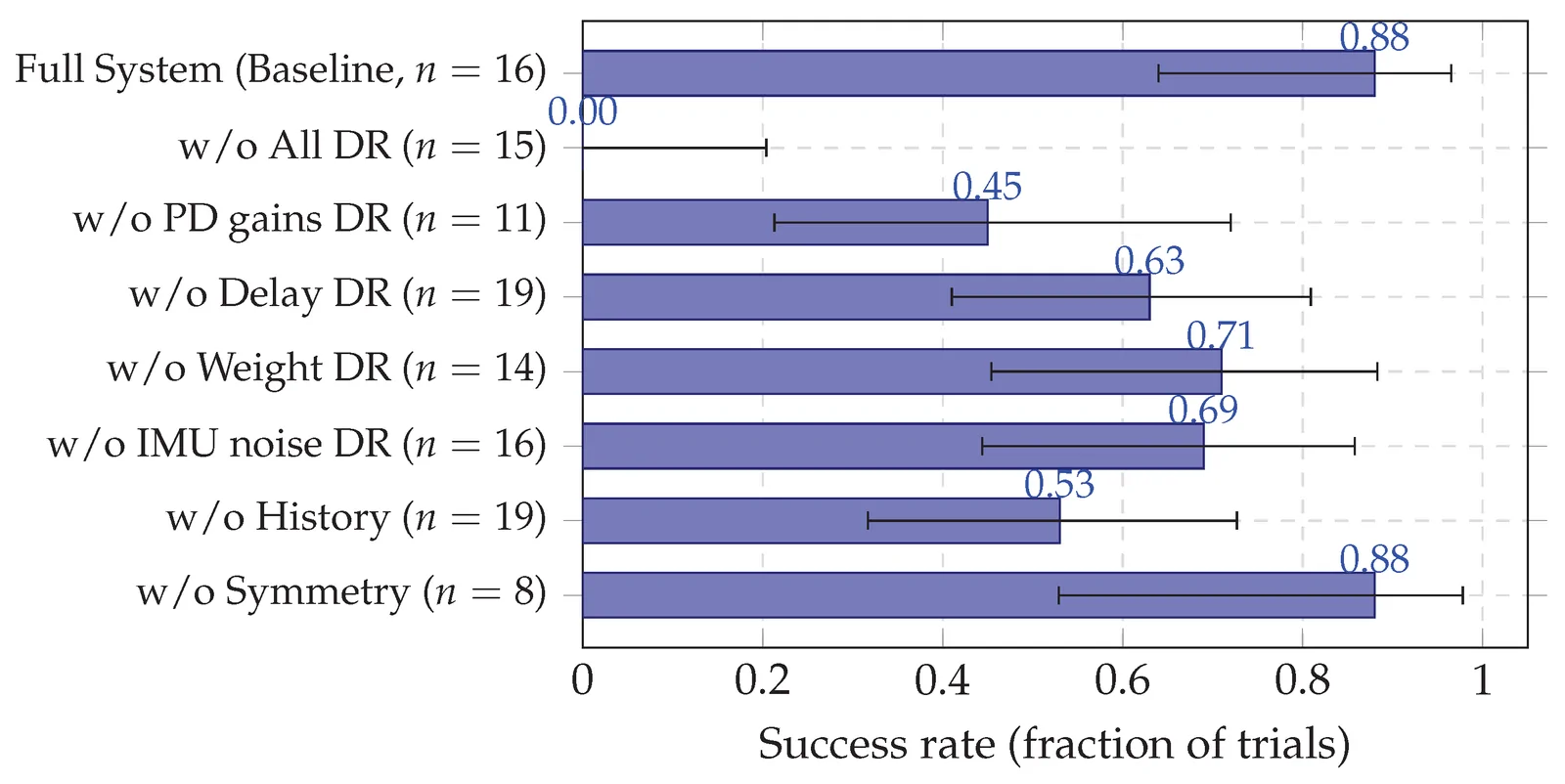
Ablation study on the free-driving scenario (not the license-test corridor). Success rates are measured on the real robot and scooter. Each configuration was retrained from scratch with a single random seed, so differences between configurations with similar success rates are not separated from run-to-run training variability. Every configuration was trained for 8 h and evaluated under the same protocol and success criterion as the standard driving trials. Error bars are 95% binomial confidence intervals for the trial counts given in the labels.

To evaluate how individual components affect the overall system performance, an ablation study was performed on the free-driving scenario. Each ablated configuration was evaluated on the real robot and scooter, under the same protocol and success criterion as the standard scenarios of Section 4.2; the reported success rates are therefore real-world results, not simulation results. Every bar refers to the free-driving scenario alone, and its baseline is the same set of runs reported as standard free driving in Table 7. This analysis indicates which elements of the training pipeline and simulation framework had the largest effect on sim-to-real transfer in these runs. Figure 12 presents the results of removing or modifying key components from the full system baseline. The full system achieves a success rate of 0.88 with 8 h of training time. Domain randomization (denoted DR in the figure) emerges as the most critical component, with its complete removal reducing the success rate to 0.0. The non-randomized policy we tested therefore failed to transfer in every trial. Among the domain randomization parameters, PD gains and command delay show the largest effect despite extensive system identification efforts. Removing randomization of PD gains reduces performance to 0.45, while removing command delay randomization yields a success rate of 0.63. In contrast, weight and IMU noise randomization show smaller impacts, with success rates of 0.71 and 0.69, respectively, when removed.

Removing the observation history reduces the success rate to 0.53, indicating that the observation derivatives carry information relevant to maintaining balance and stability, although the confidence intervals of the two configurations overlap. As expected, symmetry augmentation does not change the final success rate (0.88), but reaches that level of performance after approximately 5.5 h of training rather than 8. Every configuration reported here was trained for the full 8 h, so this is a saving available in the training budget rather than a difference between the runs compared in Figure 12. This ablation removes both symmetry mechanisms together; the data augmentation and the reward regularization were not ablated independently, so the reported saving reflects their combined effect.

## 6. Limitations

We outline the current limitations of this study to delineate the scope of our claims. First, the system was evaluated on a single humanoid platform (Thormang3) and a single scooter model (Gogoro Viva); transfer to other robot morphologies or scooter geometries is plausible given the domain-randomization approach but is not demonstrated empirically here. Second, the operating envelope is restricted to low speeds of 0.8 to 3.0 ${m\;s}^{-1}$, capped for safety; higher-speed dynamics are not evaluated. Speed was commanded by the operator and varied across this envelope during every trial rather than being set to a fixed value; the reported success rates therefore hold over the envelope as a whole. Raising the speed cap and holding speed at commanded set points, so that success rate and roll behavior can be characterized as functions of speed, is planned for future work. Third, the learned policy controls only balance and steering: the throttle is set by the operator, and the initial launch and final stop are performed manually rather than by the policy. Achieving fully autonomous operation, including speed control and leg-based launching and stopping, is left to future work. Fourth, performance on tight-radius maneuvers remains limited, as the policy prioritizes dynamic stability over aggressive trajectory tracking, yielding lower success rates on the roundabout and the license-test corridor. Finally, quantitative results are reported over the number of trials listed for each maneuver in Table 6 and Table 7; a broader evaluation across more trials, surface types, and rider and scooter variations would further strengthen the generalization claims. Success rates are reported with 95% binomial confidence intervals, which are wide at these trial counts and overlap for several scenarios, so small differences between scenarios should not be read as meaningful. The ablation study carries a further caveat: each configuration corresponds to a single training run with a single random seed, so the difference between two configurations confounds the effect of the removed component with the run-to-run variation of training. The large effects, such as the complete failure of the non-randomized policy, are unlikely to be explained by seed variance alone, but the ordering of configurations whose success rates are close is not established by these data. In addition, the observation-history length was not swept: the ablation study compares the 20-step history against no history rather than evaluating intermediate horizons, so the sensitivity of performance to this parameter is not characterized. Domain randomization also assumes that the true real-world parameters fall within the sampled ranges, and conditions outside these ranges are not guaranteed to be handled.

## 7. Conclusions

This work demonstrates that full-sized humanoid robots can autonomously balance and steer two-wheeled vehicles through deep reinforcement learning and sim-to-real transfer under significant model uncertainty, while the heading reference, target speed, throttle, launch, and stop are presently handled externally for safety. These results provide a first proof of concept that humanoid robots can learn to operate the balance-critical dynamics of complex tools originally designed for human operation when a proper sim-to-real process is applied despite undefined dynamics, rather than a claim of full mastery across all operating conditions. Notably, the system maintains stable control in the low-speed regime of 0.8 to 3.0 ${m\;s}^{-1}$, where balance control is most challenging, achieving success rates of 0.96 on straight-line driving, 0.88 while traversing a 60 mm speed bump, and 0.73 while carrying a 70 kg passenger. Success rates are lower on maneuvers requiring sustained precise tracking, at 0.28 on the 4.5 m roundabout and 0.33 on the license-test corridor, where the policy prioritizes dynamic stability over aggressive trajectory tracking.

The integration of learned steering policies with whole-body inverse kinematics at 100 Hz demonstrates that reinforcement learning can handle the coupled dynamics of humanoid-vehicle systems under contact uncertainties and hardware degradation that were not modeled analytically in this work.

In future work we aim to improve performance on tight-radius maneuvers through lower-body control and planning with learned models. We will also implement autonomous speed control and leg-based launching and stopping.

## Figures and Tables

**Table 2:** Definition of the observations.

Symbol	Description	Source	Range	Unit
ϕ	Base roll	IMU	[−0.12,0.12]	rad
$\Delta_{\psi }$	Base yaw error	IMU + Remote	[−π,π]	rad
$\omega_{\Phi }$	Base roll angular velocity	IMU	[−1, 1]	${\mathrm{rad}\;s}^{-1}$
$\omega_{\Psi }$	Base yaw angular velocity	IMU	[−1, 1]	${\mathrm{rad}\;s}^{-1}$
θ	Scooter steering angle	Forward kinematics	[−0.5, 0.5]	rad
*v*	Base forward velocity	ORB-SLAM3 + Intel RealSense T265	[0, 5]	${m\;s}^{-1}$
*d*	IK delay	Human input	[0, 0.15]	s

**Table 3:** Noise and bias of the observations and action.

Description	Distribution	Metric	Refresh	Origin
ϕ noise	N(0,0.003)	rad	step	Datasheet + real data
ϕ bias	U(−0.02,0.02)	rad	reset	Conservative (mounting alignment)
$\Delta_{\psi }$ noise	N(0,0.003)	rad	step	Datasheet + real data
$\omega_{\Phi }$ noise	N(0,0.003)	${\mathrm{rad}\;s}^{-1}$	step	Datasheet + real data
$\omega_{\Psi }$ noise	N(0,0.003)	${\mathrm{rad}\;s}^{-1}$	step	Datasheet + real data
θ noise	N(0,0.05)	rad	step	IK precision (measured)
*v* noise	U(0,0.3)	${m\;s}^{-1}$	step	SLAM measurements
*v* bias	N(0,0.3)	${m\;s}^{-1}$	reset	SLAM measurements
*u* command noise	U(−0.05,0.05)	rad	step	IK precision (measured)
*u* command bias	U(−0.05,0.05)	rad	reset	IK precision (measured)
*x* seating pose	U(−0.1,0.1)	m	reset	Estimated, deliberately widened
*y* seating pose	U(−0.1,0.1)	m	reset	Estimated, deliberately widened
*z* seating pose	U(−0.05,0.05)	m	reset	Estimated, deliberately widened
ϕ spawn	U(−0.02,0.02)	rad	reset	Estimated (operator launch pose)
ψ spawn	U(−π,π)	rad	reset	Full range by construction
*d* command delay	U(0,15)	step	reset	Estimated (PC-to-motor stack latency)
Steering damping	U(25,200)	percentage	reset	System identification
Current speed	U(0.5,5)	${m\;s}^{-1}$	reset	Operating envelope
Gravity	U(9.32,10.3)	${m\;s}^{-2}$	reset	Engineering judgment
Rigid bodies mass	U(95,105)	percentage	reset	Engineering judgment
External push	U(−20,20)	N	reset	Engineering judgment

**Table 4:** Complete PPO training configuration.

Parameter	Value
*Network*	
Architecture	Shared-trunk actor–critic, MLP
Hidden layers	512, 512, 512
Activation	ELU
Action space	1 continuous (steering angle)
Policy distribution	Gaussian, state-independent learned σ
Observation	7 quantities × 20-step history = 140
*Optimization*	
Optimizer	Adam, adaptive learning-rate schedule
Initial learning rate	$1\times 10^{-4}$
Adaptive KL target	$2\times 10^{-3}$
Discount factor γ	0.99
GAE parameter λ	0.95
Clipping parameter ϵ	0.1
Entropy coefficient	$1\times 10^{-3}$
Critic coefficient	2.0
Bounds-loss coefficient	$1\times 10^{-4}$
Gradient clipping	1.0 (global norm)
Normalization	Observation, value, and advantage
Reward scaling	0.01
*Sampling*	
Parallel environments	4096 (2048 simulated, 2048 mirrored)
Rollout length	256 steps
Batch size	1,048,576
Minibatch size	32,768 (32 minibatches)
Epochs per update	5
*Simulation and episode*	
Simulator	Isaac Gym Preview 4 (v1.0rc4), PhysX 4.1
Simulation timestep	0.01 s (3 PhysX substeps)
Control frequency	100 Hz
Episode length	10,800 steps (108 s)
Termination	|ϕ|≥0.38 rad, or timeout
*Training run*	
Software	Python 3.8, PyTorch 1.8.1 (CUDA 11.1), rl_games 1.5.2
Hardware	Single NVIDIA RTX 3090 Ti (NVIDIA Corp., Santa Clara, CA, USA)
Wall-clock budget	8 h
Total environment steps	≈$2.0\times 10^{9}$
Throughput	≈$7\times 10^{4}$ steps/s
Independent training seeds	1

**Table 5:** Inverse-kinematics solve time for both arms, measured over 10,000 consecutive calls with the first 100 discarded, compiled with -O3. All values in μs. The control period at 100 Hz is 10,000 μs. Two cases are reported: a solve starting from the recorded initial pose, and a solve seeded with the previous solution, which is the case during continuous operation. The maximum reflects operating-system scheduling on a non-real-time kernel rather than solver behavior, so the 99th percentile is the more representative worst case.

Case	Mean	SD	Median	95th	99th	Max
From recorded start pose	149.1	119.2	90.9	415.8	476.6	1846.0
Seeded with previous solution	23.6	20.9	20.8	34.2	43.4	1157.1

**Table 6:** Success rates by maneuver type. Success rates are reported as successful trials over trials attempted, with 95% binomial confidence intervals computed by the Wilson score method.

Maneuver Type	Success Rate	95% CI	Avg. Test Duration
Straight Line	24/25 (96%)	[0.80, 0.99]	100 s
Light Turn (r=10m)	11/12 (92%)	[0.65, 0.99]	100 s
90 Deg Turn (r=3m)	13/18 (72%)	[0.49, 0.88]	10 s
Roundabout (r=4.5m)	5/18 (28%)	[0.12, 0.51]	60 s

**Table 7:** Performance on challenging scenarios. Success rates are reported as successful trials over trials attempted, with 95% binomial confidence intervals. No trial required operator intervention after the manual launch.

Scenario	Success Rate	95% CI	Notes
Speed Bump Traversal	7/8 (88%)	[0.53, 0.98]	Height: 60 mm
Dynamic Load	8/11 (73%)	[0.43, 0.90]	±70 kg passenger
License Test	3/9 (33%)	[0.12, 0.65]	15m×0.4m, >7 s
Standard Free Driving	14/16 (88%)	[0.64, 0.97]	Free driving with light turns

## Data Availability

A video demonstration of the experiments is provided directly as Supplementary Materials accompanying this article. The training environment, task and training configuration files, and the robot control code are published with this article as Supplementary Materials.

## Supplementary Materials

The following supporting information can be downloaded at: https://www.mdpi.com/article/10.3390/s26196324/s1. Video S1: Demonstration of the scooter-driving humanoid robot across the reported scenarios, including straight-line driving, turns, speed-bump traversal and passenger carrying; File S1: Source code, comprising the simulation environment, the task and training configuration files, and the robot control code.
